# Chronic Tubercular Mediastinitis: A Rare Case Presentation With Subcutaneous Emphysema

**DOI:** 10.7759/cureus.38832

**Published:** 2023-05-10

**Authors:** Sumer S Choudhary, Chetan R Khedkar, Gaurang M Aurangabadkar, Shafee M Khan

**Affiliations:** 1 Respiratory Medicine, Datta Meghe Medical College and Shalinitai Meghe Hospital and Research Centre, Nagpur, IND; 2 Department of Medicine, King Edward Memorial Hospital and Seth Gordhandas Sunderdas Medical College, Mumbai, IND; 3 Respiratory Medicine, Datta Meghe Medical College, Datta Meghe Institute of Medical Sciences, Wardha, IND

**Keywords:** virtual bronchoscopy, subcutaneos emphysema, tracheal fistula, tuberculosis, mediastinitis

## Abstract

Tuberculosis, histoplasmosis, various fungal infections, malignancy, and sarcoidosis are the most common causes of chronic or slowly progressing mediastinitis. Chronic mediastinitis of tubercular origin with subcutaneous emphysema is exceptionally uncommon, and the majority of cases are caused by trauma. Here we report the case of a 35-year-old chronic alcoholic male who presented to the Outpatient Department (OPD) with complaints of cough, chest pain, loss of weight, and intermittent low-grade fever for three months with no significant past medical history or family history for any respiratory diseases. He was admitted and all routine investigations were performed, which were normal including his chest X-ray, except erythrocyte sedimentation rate (ESR) which was raised. The patient's high-resolution Computed Tomography (HRCT) of the thorax was done which showed multiple pleural-based nodular lesions with few showing central cavitary nodules along with ground glass appearance. It also showed two fistulous tracks of 3.4-millimeter diameter, arising from the trachea at the T1 - T2 vertebral level and at the carina which led to the presence of air in the subcutaneous plane extending from the neck up to visualized abdomen suggestive of chronic mediastinitis with tracheal fistula, along with subcutaneous emphysema. This fistula was confirmed by video bronchoscopy as well as three-dimensional (3D) virtual bronchoscopy. A biopsy was taken, which was positive for acid-fast bacilli (AFB) stain, polymerase chain reaction (PCR) for tuberculosis, and positive tuberculin skin test. The patient was started on anti-tubercular treatment and on a follow-up visit upon completion of the intensive phase, his HRCT and video bronchoscopy showed fibrosing scarring with fistula closure.

## Introduction

Inflammation of the tissues in the mediastinal cavity is known as mediastinitis. It can be caused by infectious or non-infectious sources and can be acute or chronic depending on the etiology. Oesophageal perforation and open chest surgery are the most common causes of acute mediastinitis [1]. Tracheal and bronchial perforation, as well as direct infectious dissemination from the surrounding tissues, are less common causes of mediastinitis. Tuberculosis, histoplasmosis, various fungal infections, malignancy, and sarcoidosis are the most common causes of chronic or slowly progressing mediastinitis. In a small number of cases, the cause was found to be a lymphatic blockage or an autoimmune illness [1,2].

However, chronic mediastinitis of tubercular origin with mediastinal emphysema is uncommon, and the majority of cases are caused by trauma. A few case reports imply that tuberculous tracheal fistula can be adequately cured with optimum medical therapy.

## Case presentation

**Figure 1:**
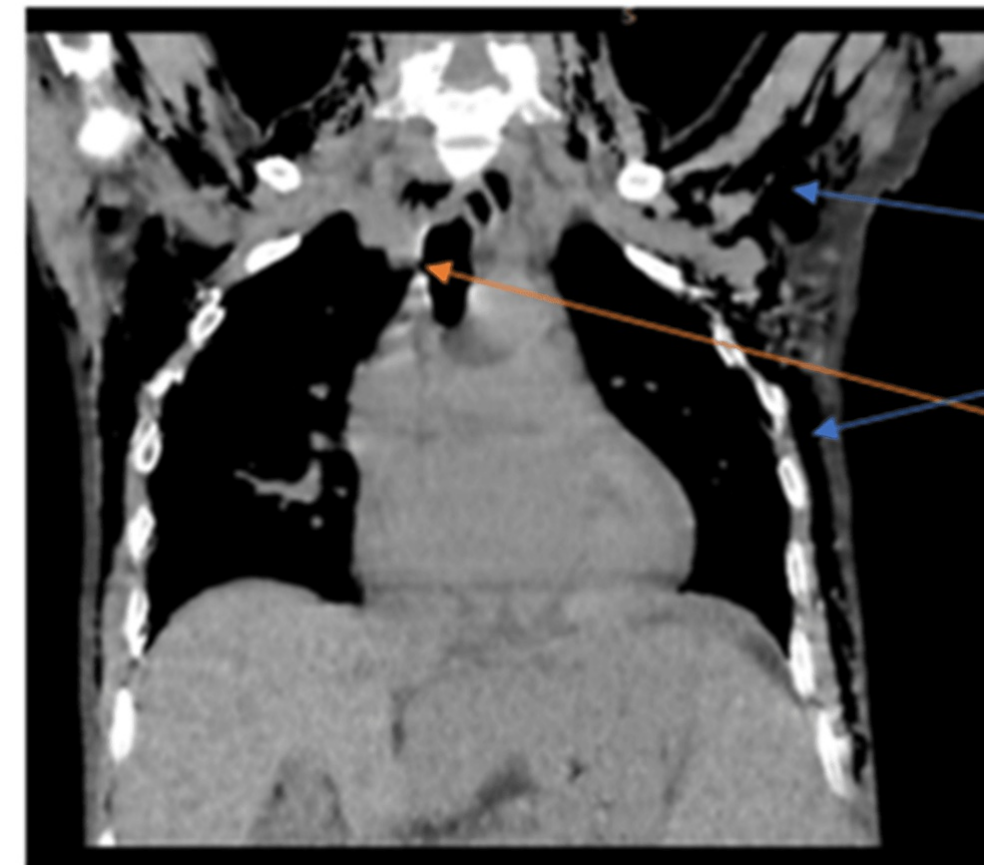
High-resolution computed tomography (HRCT) of the thorax (coronal view) showing the presence of tracheoesophageal fistula (red arrow) and subcutaneous emphysema (blue arrow)

A 35-year-old male presented to the Outpatient Department (OPD) with complaints of cough, chest pain, loss of weight, and intermittent low-grade fever for three months. The patient had no significant past or family history. He was a chronic alcoholic for more than 15 years. His initial blood counts, including total lymphocyte counts and differential lymphocyte counts, were normal. The erythrocyte sedimentation rate (ESR) was 56 millimeters/hour, and the random blood sugar, liver function tests, and renal function tests were all normal. The patient's human immunodeficiency virus (HIV) test was negative, and his chest X-ray and abdominal ultrasound were both normal. The tuberculin skin test (TST), acid-fast bacilli (AFB) smear of the lesion, and polymerase chain reaction (PCR) for tuberculosis were all found to be positive for Mycobacterium tuberculosis. A high-resolution Computed Tomography (HRCT) of the thorax was done and reported as chronic mediastinitis with tracheal fistula (Figure 1).

**Figure 2:**
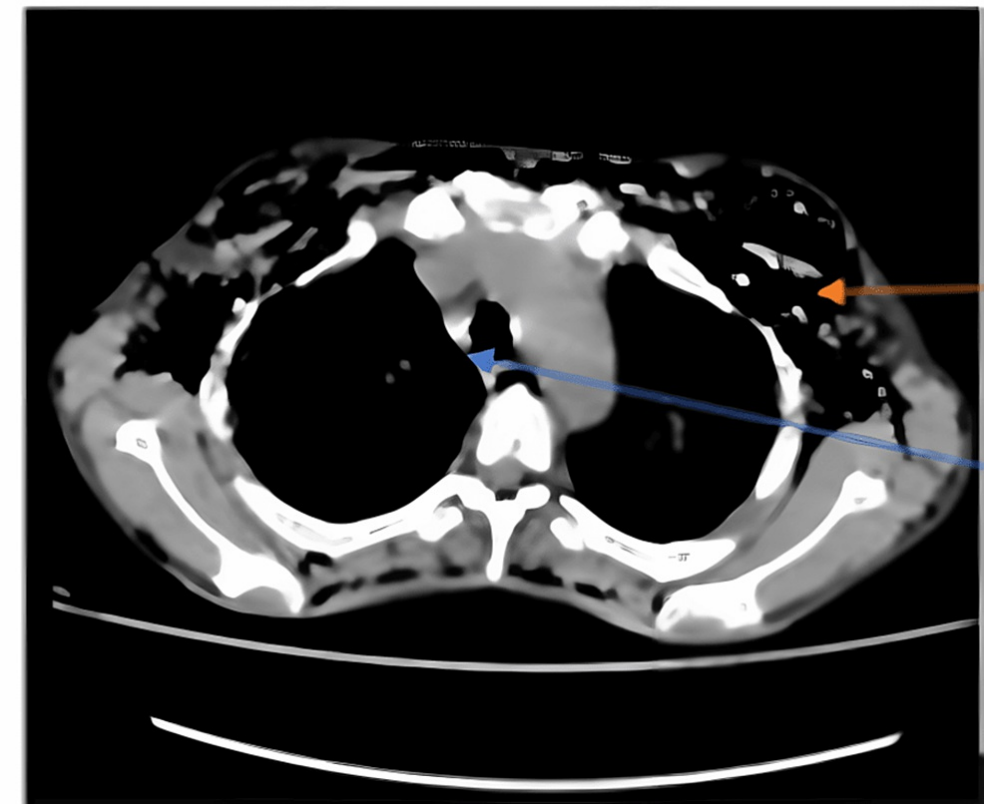
Mediastinal window showing tracheoesophageal fistula (blue arrow) and subcutaneous emphysema (red arrow)

**Figure 3:**
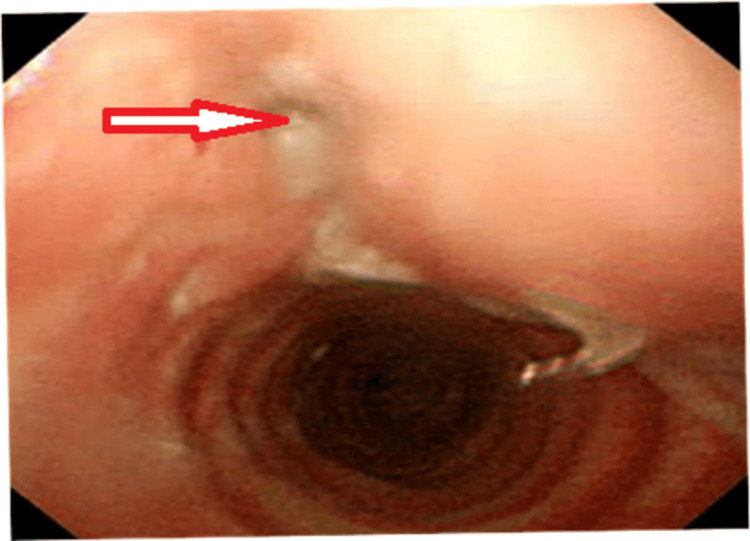
A three-dimensional (3D) virtual bronchoscopy image showing the presence of a tracheal fistula (red arrow)

It was reported as having multiple well define nodular lesions seen bilaterally averaging 9.7 mm x 8.7 mm which were peripherally distributed, with lesions showing parenchymal bands reaching up to the pleural margins. There were multiple surrounding centrilobular nodules, few of them showing linear branching patterns with a tree-in-bud appearance, and few air-space opacities. A minority of them showed central cavitary nodules. Ground glass opacities were noted in bilateral lung lobes. Multiple mediastinal sub-centimetric lymph nodes were also noted. There were two fistulous tracks with an average diameter of 3.4 mm, noted arising from the trachea at the T1 - T2 vertebral level and at the carina which led to the presence of air in the subcutaneous plane extending from the neck up to the visualized abdomen (Figure 2). Video bronchoscopy was performed and the presence of a tracheal fistula was noted. It was also confirmed on three-dimensional (3D) virtual bronchoscopy (Figure 3).

**Figure 4:**
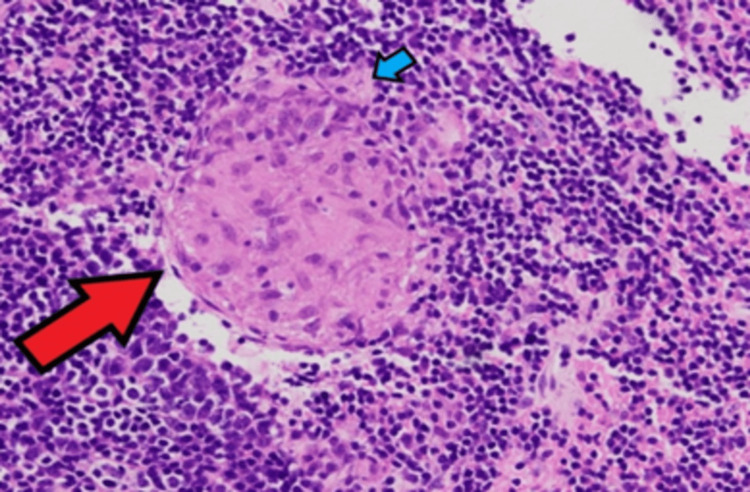
Histopathological examination (HPE) of the tracheal biopsy revealing the presence of caseous necrosis (red arrow) with acid-fast bacilli (AFB) (blue arrow)

His tracheal biopsy was done and sent for histopathological examination which showed features suggestive of chronic granulomatous infection with Ziehl-Neelsen stain was positive for Mycobacterium tuberculosis (Figure 4).

**Figure 5:**
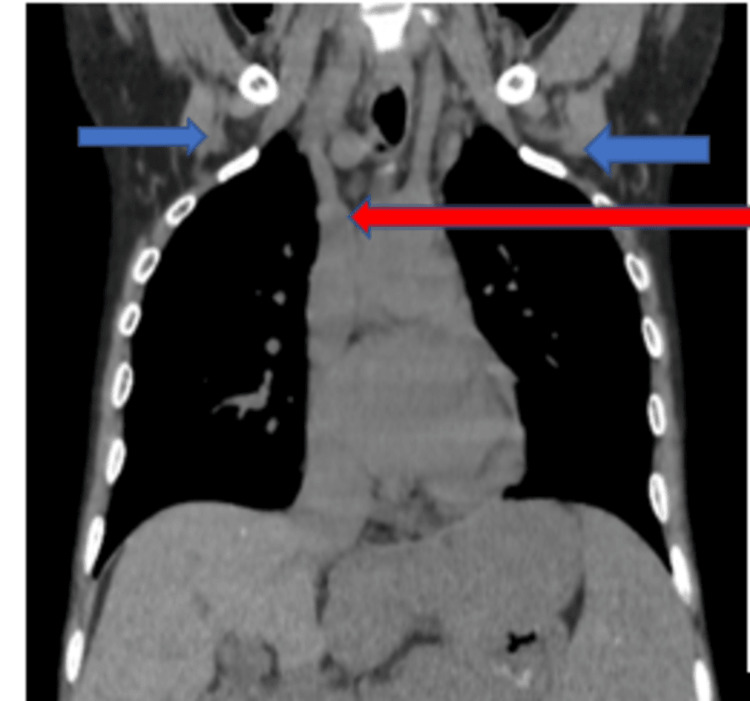
High-resolution computed tomography (HRCT) of the thorax showing healing of the tracheal fistula (red arrow) with complete resolution of the subcutaneous emphysema (blue arrow)

The patient received an antitubercular treatment regime of a total duration of six months consisting of isoniazid (H), rifampicin (R), pyrazinamide (Z), and ethambutol (E). In the follow-up visit after completion of the intensive phase of anti-tubercular therapy, the HRCT thorax revealed no evidence of a fistula and only a few nodules in the lung parenchyma (Figure 5).

## Discussion

Infections of the mediastinum are sometimes known as mediastinitis. There are two types of mediastinitis: acute and chronic. Acute necrotizing mediastinitis and post-sternotomy mediastinitis are the two main types of acute mediastinitis. Chronic mediastinitis is split into two types, namely granulomatous mediastinitis and fibrosing or sclerosing mediastinitis [3]. It has been proposed that fibrosing mediastinitis has two subtypes: localized and diffuse. The focal form usually manifests as a calcified mass in the paratracheal or subcarinal area, whereas the diffuse variant manifests as infiltrating, often non-calcified, masses encompassing various mediastinal compartments [4]. Fibrosing lesions of the mediastinum are a small but difficult category of lesions with aetiologies ranging from infectious to idiopathic to malignant [5]. Fibrosing mediastinitis manifests as a localized mediastinal mass rather than as a diffuse mediastinal illness, with the anterior mediastinal compartment being the most typically implicated [6]. Acute mediastinitis is typically caused by infections following sternotomies or by perforation of the aerodigestive tract. Acute mediastinitis caused by oropharyngeal infection dissemination, also known as descending mediastinitis, is a less common but extremely fatal type of this disease.

Chronic mediastinitis is caused by low-grade infections and in such cases the responsible organism may be fungal or mycobacterium. Because chronic infections are infrequent, very small changes in the presentation, diagnosis, and therapy of this disease have occurred. The majority are caused by a fungal disease that originates in the various mediastinal node groups, with a few being secondary to mycobacterial pathogens. The patients may be asymptomatic coming to attention following the findings of mediastinal widening on the chest radiograph. In such cases infecting organism is identified and treated with antimicrobial therapy. Chronic fungal or tubercular infections may be self-limiting, although they can progress to chronic fibrosing mediastinitis. In this patient, the HRCT scan and virtual bronchoscopy confirmed the presence of a fistula, and a biopsy was done which on histopathology confirmed tuberculosis. In most cases, cross-sectional imaging modalities are essential for diagnosis and determining the location and amount of mediastinal involvement [7]. In one of the case reports a series of three patients with diffuse pulmonary involvement had infiltrating soft-tissue masses with several dense calcification foci on Computed Tomography (CT) [8]. The CT findings are frequently adequate to imply or confirm the diagnosis of fibrosing mediastinitis, with the disease course accurately reflected In some cases, CT findings may be sufficient to rule out the necessity for diagnostic tissue samples [9]. In this patient, we started him on anti-tubercular drugs after the biopsy report. The patient showed clinical improvement and on repeat computed tomography of the thorax showed closure of the fistula which was also confirmed by bronchoscopy. Our findings indicate that "sclerosing mediastinitis" is the ultimate stage of a developing, dynamic process with morphologic manifestations similar to aberrant wound healing [10]. Thoracotomy, with the evacuation of the granulomas, is recommended, especially when the lesions are large, in order to prevent subsequent fibrosing mediastinitis with the involvement of the contiguous structures [11].

## Conclusions

Chronic mediastinitis with tracheal fistula is a rare diagnosis. There are very few clinically reported cases. High clinical suspicion in a country like India, where tuberculosis is highly prevalent, should be one of the primary differential diagnoses even if the occurrence of such patients is few. In this patient with proper investigation and confirming the diagnosis, we were able to treat the patient with medical management requiring no surgical intervention which on follow-up HRCT after completion of only the intensive phase of anti-tubercular therapy, demonstrated the closure of both of the fistula tract with resorption of the subcutaneous emphysema.

This case report highlights the varying clinical presentations of pulmonary tuberculosis with special emphasis on the sequelae and complications associated with the same. Early imaging studies such as bronchoscopy can detect complications such as tracheal fistulas.
